# Boundaries of task-specificity: bimanual finger dexterity is reduced in musician’s dystonia

**DOI:** 10.1038/s41598-024-65888-3

**Published:** 2024-07-10

**Authors:** Anna Sadnicka, Tobias Wiestler, Katherine Butler, Eckart Altenmuller, Mark J. Edwards, Naveed Ejaz, Jörn Diedrichsen

**Affiliations:** 1 Gatsby Computational Neuroscience Unit, University College London, 25 Howland Street, London, W1T 4JG UK; 2 Department of Clinical and Movement Neurosciences, University College London, London, UK; 3 Neurosciences and Cell Biology Research Institute, St George’s University of London, London, UK; 4 Faculty of Health, School of Health Professions, University of Plymouth, Plymouth, UK; 5 Division of Surgery and Interventional Science, University College London, London, UK; 6 London Hand Therapy, Mayo Clinic Healthcare, London, UK; 7 Hannover University of Music, Drama and Media, Hannover, Germany; 8 Institute of Psychiatry, Psychology and Neuroscience, King’s College London, London, UK; 9 Western Institute of Neuroscience, University of Western Ontario, London, Canada

**Keywords:** Task-specific dystonia, Musicians’ dystonia, Dexterity, Motor control, Finger individuation, Neuroscience, Neurology

## Abstract

Task-specific dystonia leads to loss of sensorimotor control for a particular motor skill. Although focal in nature, it is hugely disabling and can terminate professional careers in musicians. Biomarkers for underlying mechanism and severity are much needed. In this study, we designed a keyboard device that measured the forces generated at all fingertips during individual finger presses. By reliably quantifying overflow to other fingers in the instructed (enslaving) and contralateral hand (mirroring) we explored whether this task could differentiate between musicians with and without dystonia. 20 right-handed professional musicians (11 with dystonia) generated isometric flexion forces with the instructed finger to match 25%, 50% or 75% of maximal voluntary contraction for that finger. Enslaving was estimated as a linear slope of the forces applied across all instructed/uninstructed finger combinations. Musicians with dystonia had a small but robust loss of finger dexterity. There was increased enslaving and mirroring, primarily during use of the symptomatic hand (enslaving *p* = 0.003; mirroring *p* = 0.016), and to a lesser extent with the asymptomatic hand (enslaving *p* = 0.052; mirroring *p* = 0.062). Increased enslaving and mirroring were seen across all combinations of finger pairs. In addition, enslaving was exaggerated across symptomatic fingers when more than one finger was clinically affected. Task-specific dystonia therefore appears to express along a gradient, most severe in the affected skill with subtle and general motor control dysfunction in the background. Recognition of this provides a more nuanced understanding of the sensorimotor control deficits at play and can inform therapeutic options for this highly disabling disorder.

## Introduction

Skilled individuated finger movement represents one of the pinnacles of human development. However, in a small proportion of people, a deficit of motor control specific to a motor skill emerges, called task-specific dystonia. This painless loss of coordination often results in the abnormal posturing of individual fingers whilst performing the specific task. The highest relative prevalence is seen in musicians^1^. Although focal in nature, these disorders are hugely disabling and in musicians frequently terminate a professional career^2^.

Clinical features of task-specific dystonia point to a sensorimotor system that is failing to co-ordinate the exquisite spatiotemporal demands required for the neural control of skilled movements, especially when pushing the system to the performance limit^3–8^. There is often loss of the normal selectivity of muscles with associated overflow of muscle contractions into ipsilateral accessory muscles that are not usually used to perform the task^6–10^. Additionally when task-specific dystonia affects the hand, some patients show *mirror dystonia*, defined as involuntary movements resembling dystonia in the symptomatic hand that occur when the contralateral, *asymptomatic* hand moves^11^. This is distinct to mirror movements, unintentional movements of one side of the body that mirror intentional movements on the opposite side and though not classically associated with task-specific dystonia, can be seen in people with dystonia in general^9^. In the task-specific context (e.g. whilst playing the instrument), behavioural analysis reveals specific deficits for each individual^12,13^. However, at the group level, diagnostic biomarkers are sorely needed so that we can better categorise patients, understand mechanism and quantify severity.

**Figure 1:**
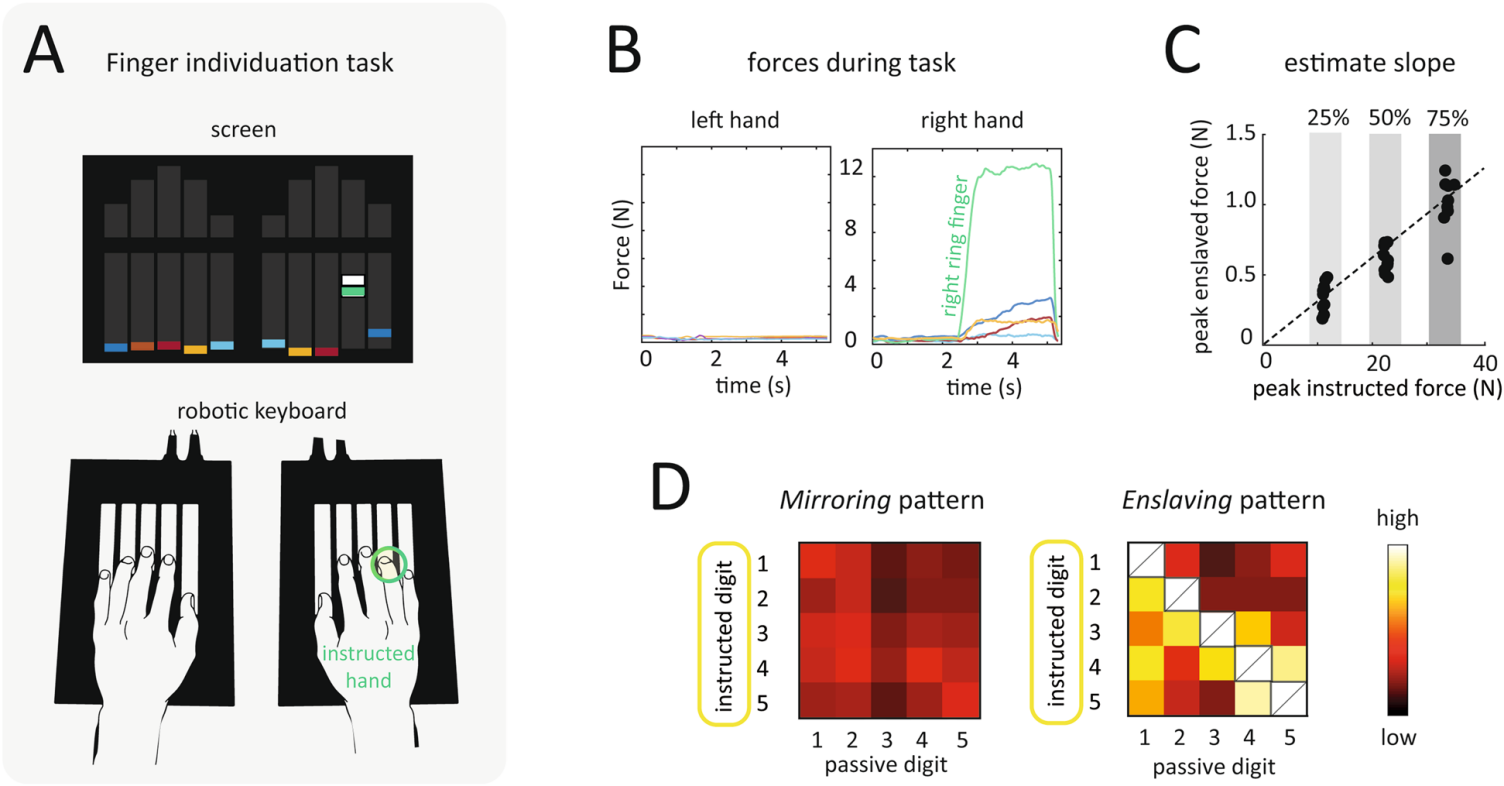
Behavioural assessment of finger individuation. (**A**) Participants placed both hands onto a custom keyboard-device that measured the isometric forces generated at the fingertips during finger presses. Participants were instructed to generate isometric flexion forces with the instructed finger to match 25%, 50% or 75% of maximal voluntary contraction for that finger. (**B**) Force trajectories produced by an exemplary patient during a single finger press showing enslaved forces in the uninstructed fingers of the same hand as well as mirrored forces in the fingers of the opposite hand. (**C**) Linear-slopes were estimated for all instructed/uninstructed finger combinations to obtain the enslaving and mirroring patterns. Linear-slopes were estimated for all instructed/uninstructed finger combinations to obtain the enslaving and mirroring patterns. (**D**) The resulting enslaving pattern and mirroring pattern are shown with a scaled colour weighting from low (dark red) to high (white).

In this study we used a finger individuation task to explore finger dexterity in healthy musicians and musicians with dystonia^14,15^. The task assessed the ability to individuate finger movements and reliably quantified any overflow of movement to other fingers in the instructed hand (enslaving) and to the contralateral hand (mirroring) (Fig. 1). As motor training is known to improve finger individuation^14^, we expected musicians to show especially good performance on this task. We were therefore interested in whether this task could reliably differentiate between musicians with and without dystonia. We were also interested in whether the abnormalities would provide a specific behavioural signature that related to the clinically affected fingers. The latter part of our analysis therefore focused on finger independence across combinations of finger pairs.

## Results

The task instructions involved making isolated presses with just the instructed finger while keeping forces in all other fingers in either hand as low as possible. These individuated finger presses resulted in undesired enslaved forces in the uninstructed fingers of the same hand as well as mirrored forces in the fingers of the other hand. Therefore, higher *enslaved and mirrored forces* indicated a lower ability to individuate fingers movements (Fig. 1).

### Reliability of enslaving and mirroring within individuals

Firstly, we confirmed that the patterns across fingers were highly reliable within individuals by calculating the split-half reliability. The data was divided into odd and even blocks, estimated for each half separately, and then the 20 values were correlated across the two halves. The average split-half correlation for enslaving for controls was r = 0.945 (95% confidence interval, 0.912–0.966) and for patients was r = 0.965 (0.951–0.975). Mirroring patterns were also highly reliable, with split-half correlations for controls being r = 0.827 (95% confidence interval, 0.773–0.869) and for patients r = 0.824 (0.753–0.876).

### Enslaving

**Figure 2:**
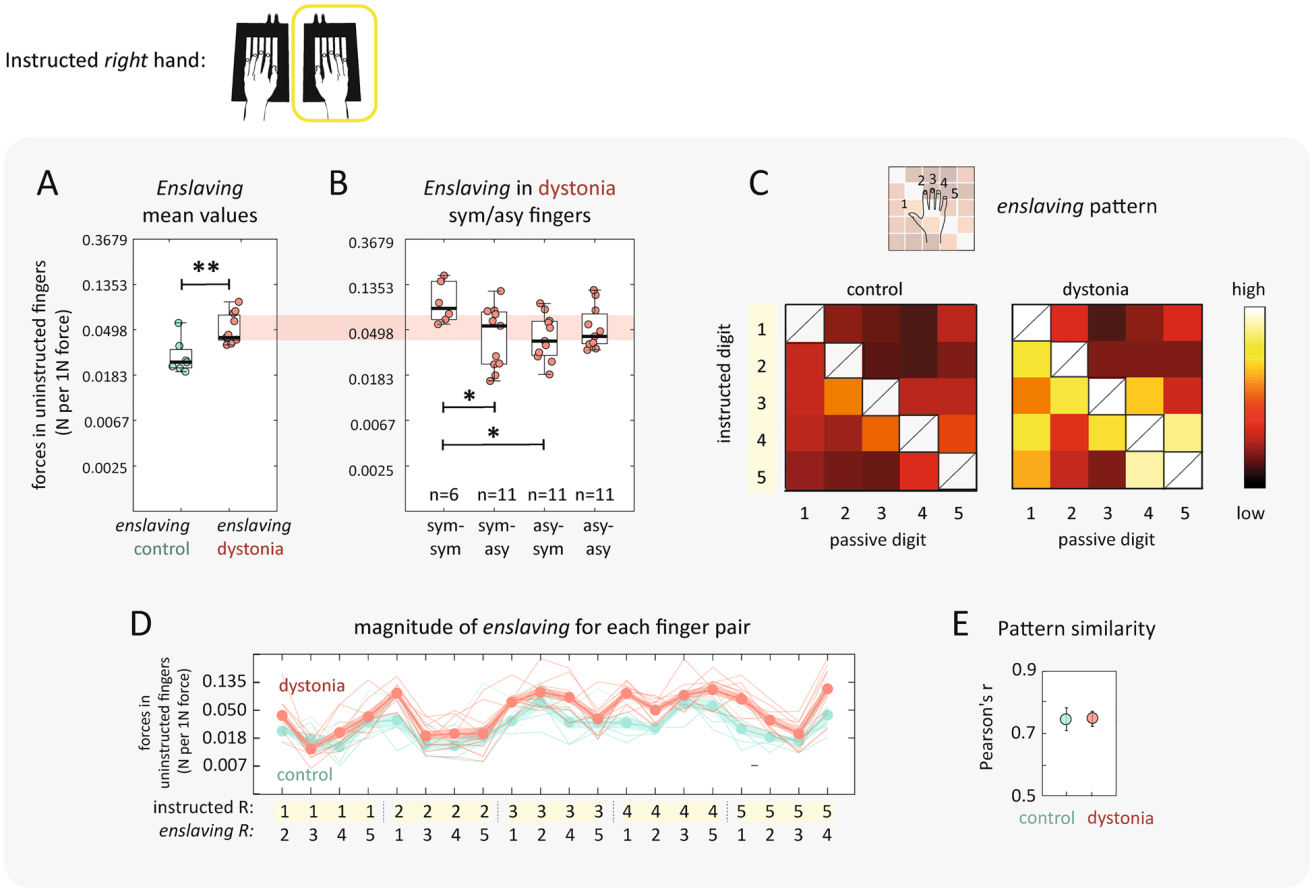
Enslaving during right hand finger presses. Green = control. Red = dystonia. (**A**) Individuated finger presses resulted in undesired enslaved forces in the fingers of the same hand. Mean values across all finger pairs were significantly higher in musicians’ with dystonia. Individual data points are scattered within box plots which median and interquartile ranges shown. Whiskers encompass non-outlier minima and maxima data points. (**B**) All musicians with dystonia were symptomatic in the right hand and a breakdown of enslaving for different combinations of symptomatic (sym) versus asymptomatic (asy) is plotted (statistical comparisons detailed in text). In (**C**) and (**D**) enslaving patterns in the right hand across all possible combinations of instructed/uninstructed finger pairs are visualised. (**C**) **|** Scaled values are shown over a colour range within a 5 × 5 matrix. The diagonal is crossed out as the instructed finger cannot be enslaved to itself. (**D**) **|** Individual values are plotted with thin lines and the group mean is indicated by connected circular markers with shading to indicate the standard error. (**E**) **|** In order to statistically compare pattern similarity we calculated Pearson’s correlations between enslaving and mirroring patterns for patients and controls using a cross-validated approach (see methods). Patterns were indistinguishable between the two groups. All statistical results are reported in the text and asterisks indicate level of significance: *p < 0.05, **p <  < 0.001.

Enslaved forces were significantly larger in musicians with dystonia than for healthy musicians. This effect was especially clear during individuated finger presses with the *right symptomatic* hand. In patients, 1N of force applied by the instructed finger in the right hand resulted in 0.046N of enslaved forces in the uninstructed fingers of the same hand. In comparison, enslaved forces for right-handed finger presses in healthy musicians were significantly smaller at 0.028N/1N of enslaved forces (Fig. 2A; t_16_ = 3.56, *p* = 0.003). For musicians with dystonia, a sub-analysis of symptomatic versus asymptomatic finger combinations revealed subtle differences (Fig. 2B, one-way ANOVA F (3,35) = 3.14, *p* = 0.038, eta-squared 0.212). For the six patients that had more than one finger affected in the right hand, enslaving was higher across symptomatic fingers (post hoc: ‘sym-sym’ to ‘sym-asy, *p* = 0.042; ‘sym-sym’ to ‘asy-sym’, *p* = 0.041). Other finger group comparisons were not significant (*p *> 0.05). Whilst this sub-analysis relied on a smaller cohort of musicians with dystonia (n = 6), and is a modest effect, it does associate an exaggeration of increased enslaving to fingers that are co-implicated in dystonia symptomatology.

**Figure 3:**
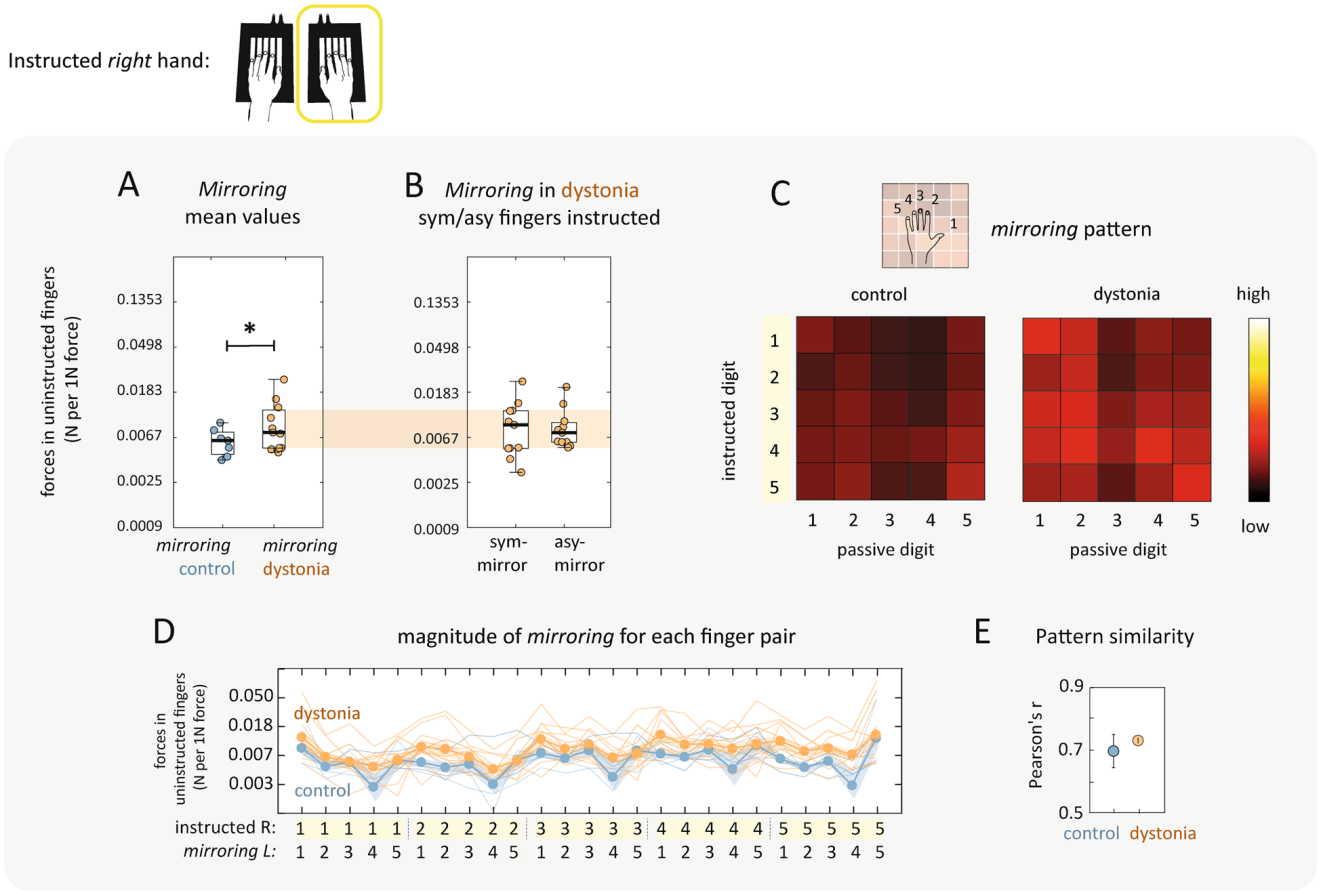
Mirroring during right hand finger presses. Blue = control. Orange = dystonia. (**A**) Individuated finger presses resulted in undesired mirroring forces in the fingers of the contralateral hand. Mean values across all finger pairs were significantly higher in musicians with dystonia. Individual data points are scattered within box plots which median and interquartile ranges shown. Whiskers encompass non-outlier minima and maxima data points. (**B**) All musicians with dystonia were symptomatic in the right hand and had similar levels of mirroring when either symptomatic (sym-mirror) or asymptomatic (asy-mirror) fingers in the right hand were instructed (null statistical comparisons detailed in text). In (**C**) and (**D**) mirroring patterns in the left hand across all possible combinations of instructed/uninstructed finger pairs are visualised. (**C**) **|** Scaled values are shown over a colour range within a 5 × 5 matrix. (**D**) **|** Individual values are plotted with thin lines and the group mean is indicated by connected circular markers with shading to indicate the standard error. (**E**) **|** Pearson’s correlations revealed that the mirroring patterns for patients and controls were indistinguishable. All statistical results are reported in the text and asterisks indicate level of significance: *p < 0.05.

**Figure 4:**
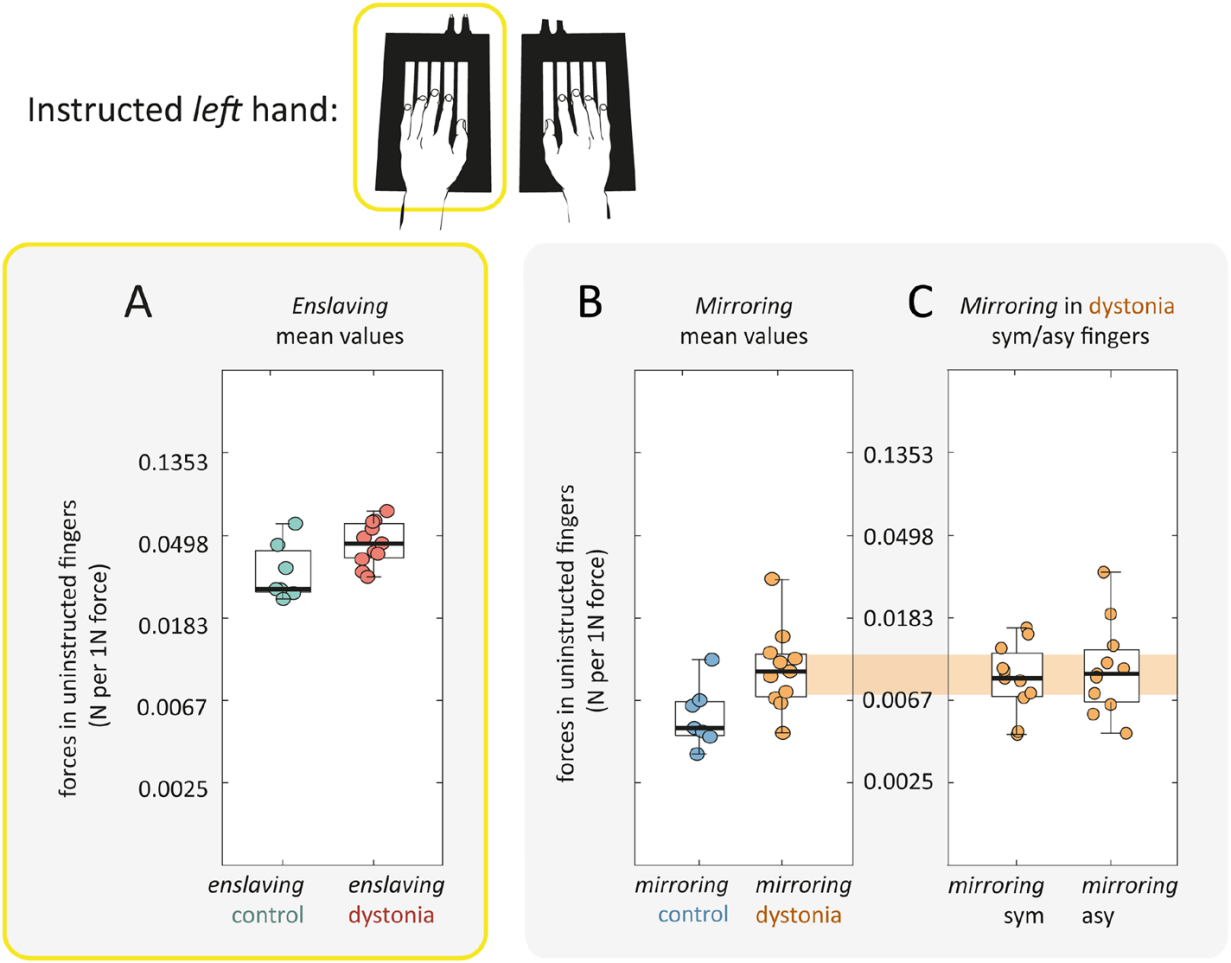
Increases in mean enslaving and mirroring in musicians with dystonia more subtle when left hand instructed. Individual data points are scattered within box plots which median and interquartile ranges shown. Whiskers encompass non-outlier minima and maxima data points. A) mean enslaving, B) mean mirroring, C) mirroring in symptomatic (sym) versus asymptomatic (asy) fingers.

We then looked at the detailed *pattern* of enslaved forces across all instructed/uninstructed finger combination to assess whether musicians with dystonia had a significant departure from the average pattern architecture in healthy musicians (Fig. 2C-E). To quantify pattern similarity, we estimated the correlation between enslaving between patients and controls using a cross-validated approach (see methods). Enslaving patterns for individual patients were highly correlated with the average control pattern (r = 0.726), and this correlation was on par with how much each individual control pattern correlated with their group’s average pattern (Fig. 3E, t_16_ = 0.64, *p *= 0.530). During *left hand* presses, musicians with dystonia also demonstrated slightly larger enslaved forces in comparison to controls. The effects reported in the left-hand were however weaker to those reported above for the right-hand (Fig. 4A, enslaving, t_16_ = 2.10, *p *= 0.052).

### Mirroring

During *right hand* finger presses, we characterised involuntary force changes on the contralateral non-instructed hand. Overall, the mirrored forces observed during presses with the right hand were significantly larger in patients than in healthy musicians (Fig. 3A; patients: 0.009N/1N; controls: 0.006N/1N; t_16_ = − 2.70, *p *= 0.016). This effect did not appear to be dependent on whether the instructed finger was symptomatic or asymptomatic (Fig. 3B; t_10_ = − 0.142, *p *= 0.89). While correlations for mirroring patterns were overall lower, mirroring patterns for individual patients and individual controls were equally correlated with the average mirroring pattern for controls (Fig. 3C-E, t_16_ = 0.505, *p *= 0.618). During *left hand* presses, musicians with dystonia also demonstrated slightly larger mirrored forces in comparison to controls during (Fig. 4B, mirroring, t_16_ = 2.01, *p *= 0.062). We did not find evidence of a behavioural correlate of mirror dystonia as the magnitude of mirroring was similar in dystonic fingers and non-dystonic fingers when the left asymptomatic hand was instructed (Fig. 4C; t_10_ = − 0.716, *p *= 0.481).

Taken together, the results show that patterns of enslaving and mirroring are highly reliable within individuals. On average both enslaved and mirrored forces in patients were larger than in control musicians, revealing a systematic upregulation in both enslaving and mirroring in musicians with dystonia. Enslaving was further exaggerated when more than one finger was affected in the right hand across symptomatic fingers.

## Discussion

Our results provide evidence that musicians with dystonia demonstrate subtle, bilateral increases in enslaving across fingers even during a task removed from musical performance. This effect was particularly evident during individuated finger presses with the symptomatic hand and was exaggerated when both the instructed and enslaved finger were affected. The overall pattern of finger independence across all combinations of finger pairs, however, remained remarkably similar for patients and controls. We also found evidence of increased mirroring of the contralateral hand.

The observed increase in enslaving and mirroring across fingers in this study are at least partly neural in origin. While abnormal enslaved forces within a hand could be caused by a specific anatomical difference in people with musician’s dystonia, this explanation is not tenable for mirroring *across* hands. Evidence for a neural component also comes from the fact that finger enslaving can be reduced by practice^14^, and it is substantially larger following damage to the motor circuits that control hand function^15^. Interestingly, when the patients and controls in this study performed a single finger press task in a related functional MRI study, the representation of individual fingers in primary sensory and primary motor areas was equivalent to controls^16^. Thus, the increased enslaving and mirroring observed in this paradigm does not have a known neural correlate in primary sensorimotor areas.

As we have only measured individuation at a single time point after symptoms have developed its mechanistic significance is uncertain. For example, increased enslaving and mirroring could be a relevant *vulnerability* that in combination with other risk-factors contributes to the *development* of task-specific dystonia. Alternatively, increased enslaving and mirroring could by a *byproduct* of the primary deficit, evidence of an upregulated sensorimotor control system that is trying to *compensate* for dystonic deficits. Either way the finding that multiple fingers activate when a single finger press is intended, is significant in a disorder in which the fine control of dexterity is impaired and is a potential biomarker.

**Figure 5:**
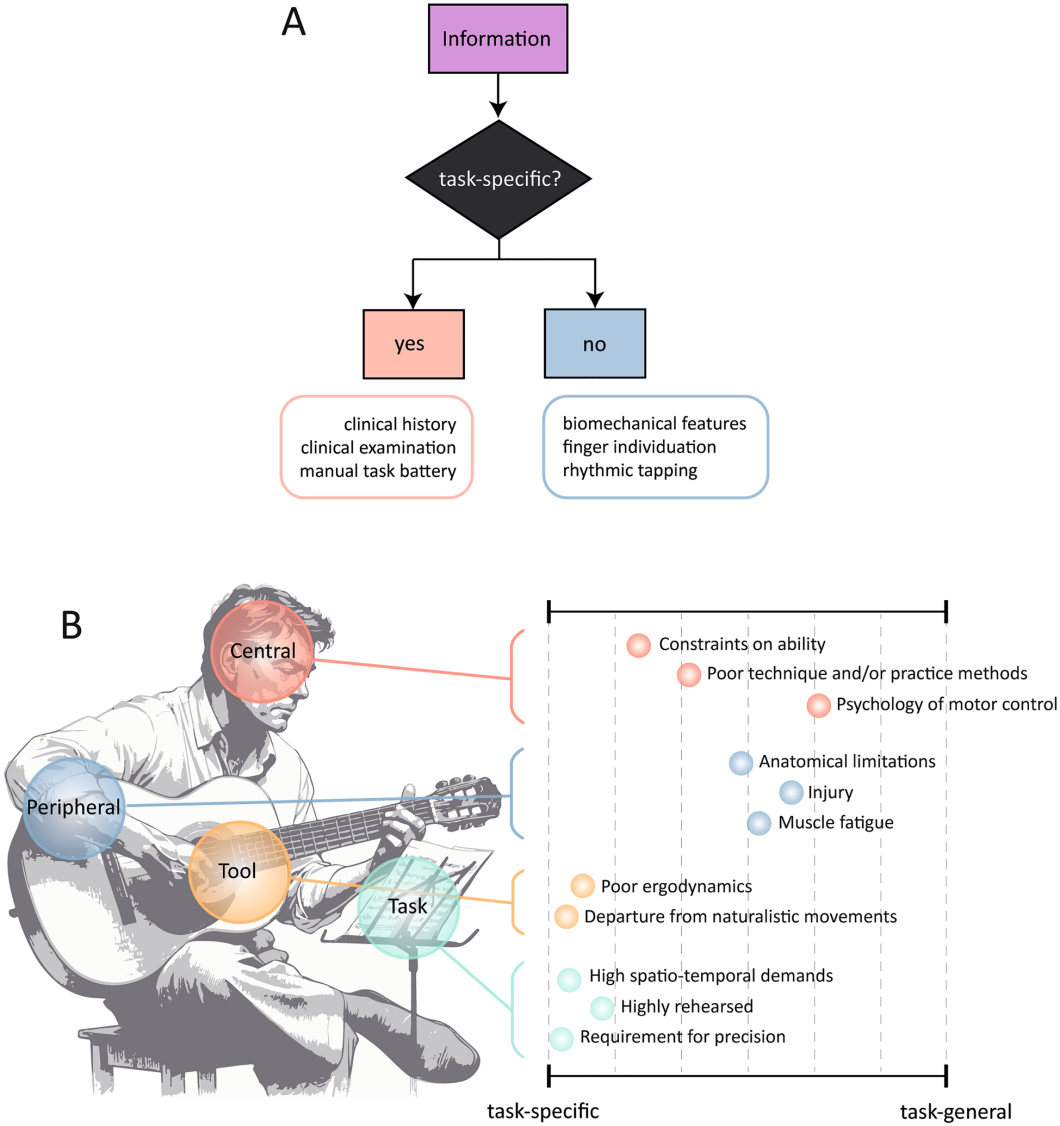
Depending on the information informing the decision a patient may or not be classed as having a task-specific deficit making this a false dichotomy. For example, at presentation the patient and clinician often only identify a single affected task and extended testing of manual tasks in the lab may also reveal a selective deficit^18^. However, in the background there are multiple neural and biomechanical that are task-invariant^6–8^. (**B**) The associated task remains important as it defines specific risk factors and perpetuating factors. Depending on the profile of different features for each individual different therapeutic interventions can then be suggested. Risk factors also range in whether they are task-specific or more general.

Our results flag certain difficulties with the name *task-specific* as we found a *bilateral* reduction in finger individuation in a related but distinct task to musical performance. Specifically, we have found upregulated *mirror movements* (*voluntary* unilateral movement of a limb that cause *involuntary* movement or mirroring of the homologous muscles of the opposite limb), a phenomena that more commonly associated with other congenital and acquired disorders such as idiopathic Parkinson’s disease and Huntington’s disease^9,17^. Indeed, whether the motor deficit is task-specific or not depends on the type of information or measure used to assess this (Fig. 5A). For example, at presentation the patient and clinician often only identify a single affected task, and extended testing of a range of manual tasks in the lab also supports a task-specific deficit in many^18^. However, in the background many task-general features are also associated with the disorder. For example, *bilateral* abnormalities hand biomechanics (such as limited active and passive range of finger abduction) have been documented since the 1990s^19^. Task-general abnormalities across multiple neural regions have also been documented experimentally^6,20–22^. Given our data and these observations we believe it is more accurate to describe musician’s dystonia as a disorder that expresses along a gradient, most severe in the affected skill with other objective abnormalities of the sensorimotor system in the background. Task-specific and task-general is likely a continuum. This may also explain why with time, many individuals experience difficulties when the symptomatic hand is used for other tasks such as playing a second instrument, writing or typing^23–25^. Previously known as complex task-specific dystonia, this spread of motor dysfunction shows how task-specific boundaries can readily break down especially if risk factors and maintaining factors are not corrected.

Despite this, there is definite utility in continuing to consider the fact the motor impairment is associated with a specific skill. Across populations of individuals affected with dystonia, risk factors can be identified, and many are associated with the specific task (Fig. 5B). For example, the highest relative prevalence in professional musicians suggests that especially greatly rehearsed skills are high risk^5^. Task-specific dystonia also preferentially involves the hand demanding the highest spatiotemporal acuity (right hand in keyboard players, left hand in players of bowed instruments). The observation that motor impairments are less frequently seen in jazz musicians is thought to relate to the flexibility of timing and sequences intrinsic to this music form^5,26,27^. Tools with poor ergo dynamics are associated with high prevalence, as exemplified by 10% of telegraphists that developed dystonia when communicating morse code^28^. Identifying such risk factors furnishes us with strategies for how to prevent the disorder and also how to tailor treatment to address modifiable risk factors. Furthermore, rehabilitation methods that are task-specific, such as sensorimotor retraining and differential learning, can be highly efficacious^29^. By retraining and re-injecting variability into movement repetitions, one case series of 40 patients achieved complete recovery in 80%^29^.

Limitations of this study include a small number of patients, that almost all were male and that by chance symptomatic musicians were all right-handed (control group matched). We also only studied pianists and guitarists (rather than a range of instrumentalists) and did not study other subtypes of task-specific dystonia such as writing dystonia. Studies with greater variability of patient and control characteristics will therefore inform future discussions about when our findings of increased enslaving and mirroring can be generalised. A remaining priority is also to better establish the order in which documented abnormalities in task-specific dystonia occur. Most studies are conducted in musicians have had dystonia for many years and it is therefore not possible to disentangle whether abnormalities are causal or compensatory. However, if vulnerability factors can be objectively identified, and a critical mass of risk factors are quantified, preventative strategies could be put in place to protect against dystonia. Many music colleges are now equipped with performance labs that allow a wide range of physiological and neural processes to be documented. Although highly speculative, it may be that the magnitude of enslaving and mirroring is useful metric towards this purpose (*susceptibility* biomarker). If an individual starts to show increasing enslaving and mirroring, training to reduce this could be protective. Alternatively, it could be delineating feature of task-specific dystonia with use as a composite *diagnostic* biomarker (in combination with other factors). Equally, if increased enslaving and mirroring turns out to be a consequence that is apparent only after disease onset, changes in enslaving magnitude could help capture treatment efficacy (*monitoring* biomarker). In the future, prospective studies at multiple timepoints, will start to build a fuller picture of what represents healthy musical performance and when metrics start to drift and need specific interventions.

In conclusion, our data demonstrate that the existing tendency within the motor system for enslaving and mirroring is scaled up in a disorder in which the neural control of individual finger movements is disturbed. This primarily occurred during use of the symptomatic hand. Our data inform a discussion about when and how task-specificity matters and provide a more nuanced understanding of the motor control deficits at play. Better delineation of the order in which deficits develop will help us optimise preventative and therapeutic options for this highly disabling disorder.

## Methods

### Participants

A total of 20 right-handed professional musicians took part in the study, 11 with dystonia and 9 without. All musicians fulfilled the following inclusion criteria: (1) had completed postgraduate musical training; (2) performed either as a soloist or ensemble player; (3) musicianship was their primary source of income. The patient group consisted of 11 musicians (10 male; mean age = 49.9 years, SD = 7.85). Patients were recruited via clinics at the National Hospital for Neurology and Neurosurgery and London Hand Therapy. Two neurologists (MJE and AS) independently confirmed the diagnosis. For each patient, symptomatic fingers during musical performance with their primary instrument of choice were noted (guitar or piano). Symptomatic fingers were defined as (1) reported to be affected by patients and (2) had an objective deficit of motor control on examination by specialist (such as an abnormal posture, or recurrent pattern of abnormal movement on action). All patients had dystonic symptoms in the right-hand whilst playing and two patients had bilateral symptoms (Table 1). The severity of overall impairment for each individual was quantified using the Tubiana and Chamagne scale^30^. The control group consisted of nine healthy musicians with no history of musculoskeletal/functional impairment of the upper limbs (all male; mean age = 41.0 years, SD = 14.54). Participants in this study also took part in a separate fMRI study which has been published previously^16^. The groups were matched for age (t_18_ = 1.75, *p *= 0.097, unpaired t-test). Data were excluded for two controls due to equipment failure. Written consent was obtained from each participant and all methods were performed in accordance with the relevant guidelines and regulations. The local ethics committee approved all study procedures (South Central-Oxford B Research Ethics Committee, IRAS ID233848).

**Table 1:** Demographic and clinical details of final patient group.

Age	Gender	Instrument	Symptomatic digits	Duration	Severity
39	Male	Piano	Right thumb	2	3
49	Male	Piano	Right and left thumbs	5	2
56	Male	Guitar	Right index	9	2
49	Male	Piano	Right middle and ring; left thumb	7	3
68	Male	Piano	Right ring and little	26	2
51	Male	Guitar	Right thumb and index	6	2
51	Female	Guitar	Right index, middle and ring	3	2
39	Male	Guitar	Right middle	2	3
47	Male	Piano	Right ring and little	4	4
51	Male	Guitar	Right middle and ring	6	3
49	Male	Guitar	Right thumb	16	2

Mean duration of dystonic symptoms was 7.82 years (SD = 7.21). All patients had symptoms in the right-hand and two also had dystonia of the left thumb. The Tubiana-Chamagne scale (TCS) has the following possible values: 0-unable to play, 1-plays several notes but stops because of blockage or lack of facility; 2-plays short sequences without rapidity and with unsteady fingering; 3-plays easy pieces but is unable to perform more technically challenging pieces; 4-plays almost normally, difficult passages are avoided for fear of motor problems; 5-returns to concert performances). The average Tubiana-Chamagne score for the cohort was 2.55 (SD = 0.69).

### Task

Participants were seated comfortably in front of a computer display with their hands resting on piano-like keys of a custom-built device (Fig. 1A). Force transducers (Honeywell FS Series, dynamic range 0–25N) underneath each piano key continuously measured forces applied by that finger. The resulting forces generated across all 10 fingers during each trial were digitised at 200 Hz and used for subsequent analysis. The individuation assessment began with two measurements of the maximum voluntary force on each finger^15^. All subsequent trials required participants to produce isometric flexion forces at a percentage (25%, 50%, and 75%) of the instructed finger’s maximum voluntary force. Each trial began with a short preparation phase (2-3s), during which a force target-zone (target force ± 25%) was presented in green on a screen in front of the participant for a single finger (Fig. 1A). Following this go-cue, participants performed an isometric press with the instructed finger to match and maintain the target-zone for 2–3s. Participants were asked to keep the non-instructed fingers of both hands on the piano-keys and minimise any force changes in these fingers. The instructed finger for each trial was chosen in pseudo-random order. Trials were grouped into blocks each consisting of 30 individual trials (3 target forces × 10 fingers). Each participant performed a total of 10 such blocks.

### Quantifying finger enslaving

Individuated finger presses with an instructed finger resulted in enslaved forces in the uninstructed fingers in the same hand (Fig. 1B)^31^. These enslaved forces increased approximately linearly as a function of the force applied by the instructed finger (Fig. 1C). As in previous papers^15,32^, we therefore quantified finger individuation ability in each participant by estimating the relationship between the forces applied in the instructed fingers and the enslaved forces in the uninstructed fingers. We did this separately for all 20 possible combinations of instructed/uninstructed finger pairs. For this analysis, the resting baseline force on each finger at the start of each trial was subtracted from the subsequent force trace of the trial. Next, for each pair of instructed finger and uninstructed finger, we plotted the peak force on the instructed finger on the x-axis and the peak enslaved force on the uninstructed finger on the y-axis (Fig. 1C). Finally, we estimated the regression line constrained to pass through the origin that best described the data points. Sensitivity to outlier data points was reduced by using robust regression with a b-squared weighting function. The resulting slope of the regression line was an estimate of finger individuation ability in each participant and quantified how much enslaved forces were produced for every N of force produced by the instructed finger. To allow for the use of parametric statistics, the regression slope was log-transformed to make it conform better to a normal distribution. The average log-slope (averaged across all finger combination) was hereafter used to as a measure of the overall degree of enslaving. The entire matrix of all 20 possible combinations of instructed/uninstructed finger pairs constituted the enslaving pattern. The patterns are visualized as a 5 × 5 matrix, which each cell representing the strength of enslaving in the uninstructed finger (column) during presses with the instructed finger (row)(Fig. 2C), or in line plots (Fig. 2D).

### Quantifying finger mirroring

Presses with the instructed finger resulted also in subtle mirrored forces in the fingers of the passive, uninstructed hand. These mirror movements during unimanual finger presses can be observed even in healthy participants, with the associated mirrored forces also increasing approximately linearly as a function of the force applied with the instructed finger/hand^33,34^. We quantified mirror movements in each participant in the same way as for enslaving, by estimating the log-slope between the peak forces in the instructed finger and the uninstructed finger in the opposing, passive hand. The log-slope was estimated independently for each of the 25 possible combinations of instructed/uninstructed fingers pairs in the active/passive hands respectively. For each participant, the log-slopes were averaged across all 25 instructed/uninstructed finger pairs to obtain a composite metric of the degree of mirroring. As for enslaving, the resulting mirroring patterns are visualized as a 5 × 5 matrix which each cell representing the strength of mirroring in the uninstructed finger in the passive hand (column) during presses with the instructed finger in the active hand (row) (Fig. 3C), or in line plots (Fig. 3D).

### Analysis of symptomatic and asymptomatic fingers (dystonia group only)

For musicians with dystonia, we compare the strength of enslaving when symptomatic fingers were involved (Fig. 2B). We classified the instructed-enslaved finger pairs as: symptomatic-symptomatic (sym-sym), symptomatic -asymptomatic (sym-asym), asymptomatic-symptomatic (asym-sym), asymptomatic-asymptomatic (asym-asym) fingers pairs. Only six musicians had more than one finger affected by dystonia (sym-sym analysis, n = 6). One-Way ANOVA compared mean values (enslaving = dependent variable, finger combination = grouping) and the eta-squared value estimated the effect size. Post-hoc multiple pairwise comparisons were performed (Tukey based on homogeneity of variances). For mirroring, when the symptomatic hand was instructed, we compared trials in which the instructed finger was symptomatic or asymptomatic (Fig. 3B). When the left asymptomatic hand was instructed, we assessed for a behavioural correlate of mirror dystonia (involuntary movements in the *symptomatic* hand that occur when the contralateral, asymptomatic hand moves)^11^. We therefore compared mirroring shown on symptomatic versus asymptomatic fingers, using a paired student’s t-test.

### Patterns of enslaving and mirroring across all combinations of finger pairs

As dystonia occurs in isolated fingers and varies in terms of which digit it effects across individuals, we wanted to examine whether musicians with dystonia had a group deviation from the normative architecture of enslaving and mirroring patterns seen in healthy musicians across all finger combinations. The enslaving/mirroring pattern for each patient was correlated with the average enslaving/mirroring pattern for all controls. The correlations were then compared to the correlation of each control pattern with the averaged pattern for the control group. To avoid a positive bias for each control comparison their individual data was removed from the averaged control pattern. The similarity between patterns for patients and controls was assessed separately for enslaving and mirroring. All correlations were Fisher-Z transformed prior to statistical testing.

## Data Availability

Summary data and analysis code to be published online at github.com/annasadnicka/dystonia_individuation.
